# Cathepsin C Regulates Cytokine-Induced Apoptosis in β-Cell Model Systems

**DOI:** 10.3390/genes12111694

**Published:** 2021-10-25

**Authors:** Tina Fløyel, Caroline Frørup, Joachim Størling, Flemming Pociot

**Affiliations:** 1 Translational Type 1 Diabetes Research, Clinical Research, Steno Diabetes Center Copenhagen, 2820 Gentofte, Denmark; caroline.froerup.01@regionh.dk (C.F.); joachim.stoerling@regionh.dk (J.S.); flemming.pociot@regionh.dk (F.P.); 2 Department of Biomedical Sciences, Faculty of Health and Medical Sciences, University of Copenhagen, 2200 Copenhagen, Denmark; 3 Department of Clinical Medicine, Faculty of Health and Medical Sciences, University of Copenhagen, 2200 Copenhagen, Denmark

**Keywords:** type 1 diabetes, lysosomal proteases, β-cell death, CTSC, MAPK, CXCL10, inflammation, pro-inflammatory cytokines, human pancreatic islets

## Abstract

Emerging evidence suggests that several of the lysosomal cathepsin proteases are genetically associated with type 1 diabetes (T1D) and participate in immune-mediated destruction of the pancreatic β cells. We previously reported that the T1D candidate gene cathepsin H is downregulated by pro-inflammatory cytokines in human pancreatic islets and regulates β-cell function, apoptosis, and disease progression in children with new-onset T1D. In the present study, the objective was to investigate the expression patterns of all 15 known cathepsins in β-cell model systems and examine their role in the regulation of cytokine-induced apoptosis. Real-time qPCR screening of the cathepsins in human islets, 1.1B4 and INS-1E β-cell models identified several cathepsins that were expressed and regulated by pro-inflammatory cytokines. Using small interfering RNAs to knock down (KD) the cytokine-regulated cathepsins, we identified an anti-apoptotic function of cathepsin C as KD increased cytokine-induced apoptosis. KD of cathepsin C correlated with increased phosphorylation of JNK and p38 mitogen-activated protein kinases, and elevated chemokine CXCL10/IP-10 expression. This study suggests that cathepsin C is a modulator of β-cell survival, and that immune modulation of cathepsin expression in islets may contribute to immune-mediated β-cell destruction in T1D.

## 1. Introduction

Type 1 diabetes (T1D) is a chronic autoimmune disease arising from a targeted immune-mediated destruction of the insulin-producing β cells resided in the pancreatic islets of Langerhans. During a local inflammation (insulitis), pro-inflammatory cytokines, including interleukin (IL)-1β, interferon (IFN)-γ, and tumor necrosis factor (TNF)-α, are secreted from invading immune cells, as well as from the β cells themselves, and mediate β-cell death and dysfunction [1,2,3]. The complex molecular signaling mechanisms and drivers of β-cell loss in T1D still remain to be fully elucidated. Crosstalk between the immune cells and the β cells, as well as endoplasmic reticulum (ER) stress and pro-apoptotic signaling are thought to play key roles in T1D pathogenesis [2,3,4]. Recently, dysfunctional autophagy caused by impaired lysosome function and leakage of lysosomal cathepsins was proposed as a contributory factor [5,6,7].

Cathepsins constitute a group of proteases originally known for their presence in the lysosomes but are now widely recognized for their functions both extracellularly and in other cellular compartments, e.g., in secretory granules, the cytosol and the nucleus [8]. The 15 known human cathepsins are classified based on their active site amino acids into serine (cathepsin A and G), aspartic (cathepsin D and E), and cysteine cathepsins (cathepsin B, C, F, H, K, L, O, S, V/L2, W, and Z/X) [9]. The cathepsins have specific and individual functions and are involved in a variety of cellular functions in addition to protein turnover in the endosomal/lysosomal compartments, including apoptosis, antigen presentation, degradation of extracellular matrix proteins as well as prohormone- and cytokine processing [8,10,11].

Dysregulation of cathepsins have been implicated in a wide array of diseases, including T1D [8,12,13,14]. Several cathepsins have been genetically associated with T1D, i.e., cathepsin H (*CTSH*), B (*CTSB*) and V (*CTSV*), and a few studies have demonstrated roles for cathepsins in regulation of β-cell function and apoptosis [5,15,16,17,18]. We previously reported that *CTSH* is downregulated by pro-inflammatory cytokines in human islets as well as in rat and human β cells, and that cathepsin H regulates β-cell function, apoptosis and disease progression in children with newly diagnosed T1D [15,19]. A recent study by Lambelet et al. indicated that cytokines impair lysosome function leading to lysosome membrane permeabilization, cathepsin B leakage and β-cell death [5]. The study showed that blocking cathepsin B activity partially protected against cytokine-induced apoptosis [5]. In addition, the cathepsin proteases have been implicated in β-cell dysfunction and death in response to known β-cell stressors in type 2 diabetes (T2D), i.e., high glucose and free fatty acids (FFA) [20,21]. Interestingly, islets from donors with T2D displayed decreased expression of cathepsin B and D [22], and the FFA palmitate caused a decrease in the expression of several cathepsins in human islets [23].

Based on the emerging experimental evidence, we hypothesized that cytokine-mediated dysregulation of cathepsin proteases contributes to β-cell apoptosis in T1D. Using different β-cell model systems, we examined the gene expression profile of the cathepsins in response to pro-inflammatory cytokines and investigated their role in cytokine-induced apoptosis. We demonstrate that cytokines modulate the expression of several cathepsins, and that cathepsin C participates in the regulation of cytokine-induced β-cell apoptosis.

## 2. Materials and Methods

### 2.1. Culture of Human Pancreatic Islets and β-Cell Lines

Human pancreatic islets were purchased from Prodo Laboratories Inc. via Tebu-Bio (donor information is available in Supplementary Table S1). Human islets were maintained in medium prepared from F-10 Nutrient Mix with GlutaMAX, supplemented with 10% heat-inactivated fetal bovine serum (FBS) and 100 U/mL penicillin, and 100 μg/mL streptomycin (All from Life Technologies, Carlsbad, CA, USA)

Upon experimental setup, islets were incubated for 24 h in medium prepared from F-10 Nutrient Mix with GlutaMAX, supplemented with 2% human serum and 100 U/mL penicillin, and 100 μg/mL streptomycin in the presence or absence of 50 U/mL recombinant human IL-1β (R&D Systems), 1000 U/mL recombinant human IFN-γ (PeproTech, Rocky Hill, NJ, USA).

The rat insulinoma INS-1E cell line [24] and the human hybrid 1.1B4 β-cell line [25] were maintained in cell culture medium prepared from RPMI-1640 with GlutaMAX, supplemented with 10% heat-inactivated FBS, 100 U/mL penicillin, and 100 μg/mL streptomycin (all from Life Technologies). The culture medium for the INS-1E cells was additionally supplemented with 50 µM β-2-mercaptoethanol (Life Technologies). Cytokine stimulation was carried out using 150 pg/mL recombinant mouse IL-1β (BD Biosciences Pharmingen) and 5 ng/mL recombinant rat IFN-γ (R&D Systems) for the INS-1E cells and 800 U/mL recombinant human IL-1β (R&D Systems), 200 U/mL IFN-γ (PeproTech) and 1000 U/mL TNF-α (R&D Systems) for the 1.1B4 cells.

Cells and islets were seeded in duplicate or triplicate in appropriate dishes and incubated in a humidified incubator at 37 °C with 5% CO_2_.

### 2.2. Transfection

Knockdown (KD) was achieved by RNA interference (RNAi) using small interfering RNAs (siRNAs) (Dharmacon, Horizon Discovery, Waterbeach, UK) targeting human *CTSC* (L-005835-00-0005; ON-TARGETplus), *CTSD* (L-003649-00-0005; ON-TARGETplus), *CTSO* (L-005843-00-0005; ON-TARGETplus), and *CTSS* (L-005844-00-0005; ON-TARGETplus); and rat *Ctsc* (M-089484-01; siGENOME) (all SMARTpools consisting of a mixture of four individual siRNAs to increase potency and specificity). A non-targeting control siRNA pool (D-001810-10-05; ON-TARGETplus) was used as a negative control. Transfection was obtained with the Lipofectamine RNAiMAX transfection reagent in Optimem medium (both from Life Technologies) as previously descried [26].

### 2.3. Gene Expression

RNA was extracted with the RNeasy Mini Kit (Qiagen, Valencia, CA, USA) or Direct-zol RNA Miniprep Kit (Zymo research, Irvine, CA, USA). The synthesis of cDNA was done using the iScript^TM^ cDNA Synthesis Kit (Bio-Rad, Hercules, CA, USA). The expression of mRNA was analyzed by real-time qPCR using TaqMan Assays and TaqMan Gene Expression Master Mix (Applied Biosystems, Waltham, MA, USA) on a CFX384 C1000 Thermal cycler (Bio-Rad). The relative expression levels were normalized to the geometric mean of three housekeeping genes (*ACTB*, *GAPDH*, *HPRT*) in human pancreatic islets or to one stable housekeeping gene (*Hprt* in rat and *GAPDH* in human cell lines) and evaluated using the 2^−ΔΔCT^ method [27].

### 2.4. Apoptosis Analyses

Apoptosis was analyzed by the measure of caspase 3/7 activity using the Caspase-Glo 3/7 Assay (Promega, Madison, WI, USA), according to the manufacturer’s protocol, and normalized to cell content using the CytoTox-Fluor Cytotoxicity Assay (Promega). Cell death was further analyzed using the Cell Death Detection ELISAplus assay (Roche, Basel, Switzerland) to detect fragmented cytoplasmic nucleosomes (DNA-histone complexes), according to the manufacturer’s protocol. Data was normalized to the DNA content; the ELISA lysates were sonicated, and the DNA measured using the QuantiFluor dsDNA Assay (Promega). Luminescence and fluorescence were measured on an Infinite M200 PRO plate reader (Tecan, Männedorf, Switzerland).

### 2.5. Immunoblotting

Cells were lysed in M-PER mammalian protein extraction reagent (Thermo Scientific, Waltham, MA, USA) supplemented with 5 mM EDTA solution and Halt protease and phosphatase inhibitor cocktail (Thermo Scientific) and centrifuged at 15,000× *g* for 10 min at 4 °C. Supernatants were collected and protein concentrations determined using the DC Protein Assay (Bio-Rad). Immunoblotting was done using Bolt 4–12% Bis-Tris Plus gels (Thermo Scientific), according to the manufacturer’s instructions. Membranes were blocked in skim milk, washed in Tris-buffered saline with 0.1% Tween 20 (TBST) and incubated in primary antibodies: anti-CTSC (#sc-74590; Santa Cruz), anti-cleaved caspase-3 (#9661), anti-c-Jun N-terminal kinase (JNK) (#9252), anti-phospho-JNK (#9251), anti-p38 (#9212), anti-phospho-p38 (#9211), anti-extracellular signal-regulated kinase (ERK) (#9102), anti-phospho-ERK (#9101), anti-C-X-C chemokine ligand 10 (Cxcl10)/Interferon γ-induced protein 10 (IP-10) (#14969), anti-immunoglobin binding protein (BiP) (#3183), anti-cytochrome C (#4272) and anti-phosphorylated inositol requiring kinase 1a (IRE1a) (#3294) (all from Cell Signaling, Danvers, MA, USA), anti-inducible nitric oxide synthase (iNOS) (#610432; BD Biosciences), anti-GAPDH (#ab9482; Abcam, Cambridge, UK), and secondary HRP-conjugated anti-mouse (#7076) or anti-rabbit (#7074) IgG antibodies (Cell Signaling). Visualization was done by chemiluminescence with LumiGLO (Cell Signaling) and a FUJI LAS4000 Imager. Quantification was done using ImageQuant TL software (GE Healthcare Life Sciences, Chicago, IL, USA).

### 2.6. NO and CXCL10 Measurements

The concentration of nitric oxide (NO) secreted by the cells into the cell culture medium was evaluated with the Griess Reagent System (Promega) nitrite assay, carried out according to the manufacturer’s protocol. The concentration of secreted CXCL10 was evaluated by Luminex xMAP technology using ProcartaPlex multiplexing assays (Invitrogen, Thermo Fisher Scientific, Waltham, MA, USA) and a MAGPIX instrument (Luminex, Austin, TX, USA) according to the manufacturer’s protocol.

### 2.7. Statistical Analysis

Data are presented as fold change or concentrations (pg/mL) with means ± SEM, unless otherwise stated. Statistical significance was determined using a two-tailed paired Student’s t-test. Cells that were transfected with cathepsin-specific siRNAs were compared with cells transfected with the non-targeting negative control siRNA, and cells that were stimulated with cytokines over a time-series were compared individually with the 0-h control condition. Results were considered statistically significant when obtaining a *p*-value < 0.05. Benjamini-Hochberg (BH) and Bonferroni corrections have been used to adjust for false discovery rates upon multiple testing.

## 3. Results

### 3.1. Cathepsin Expression and Regulation by Cytokines

**Figure 1:**
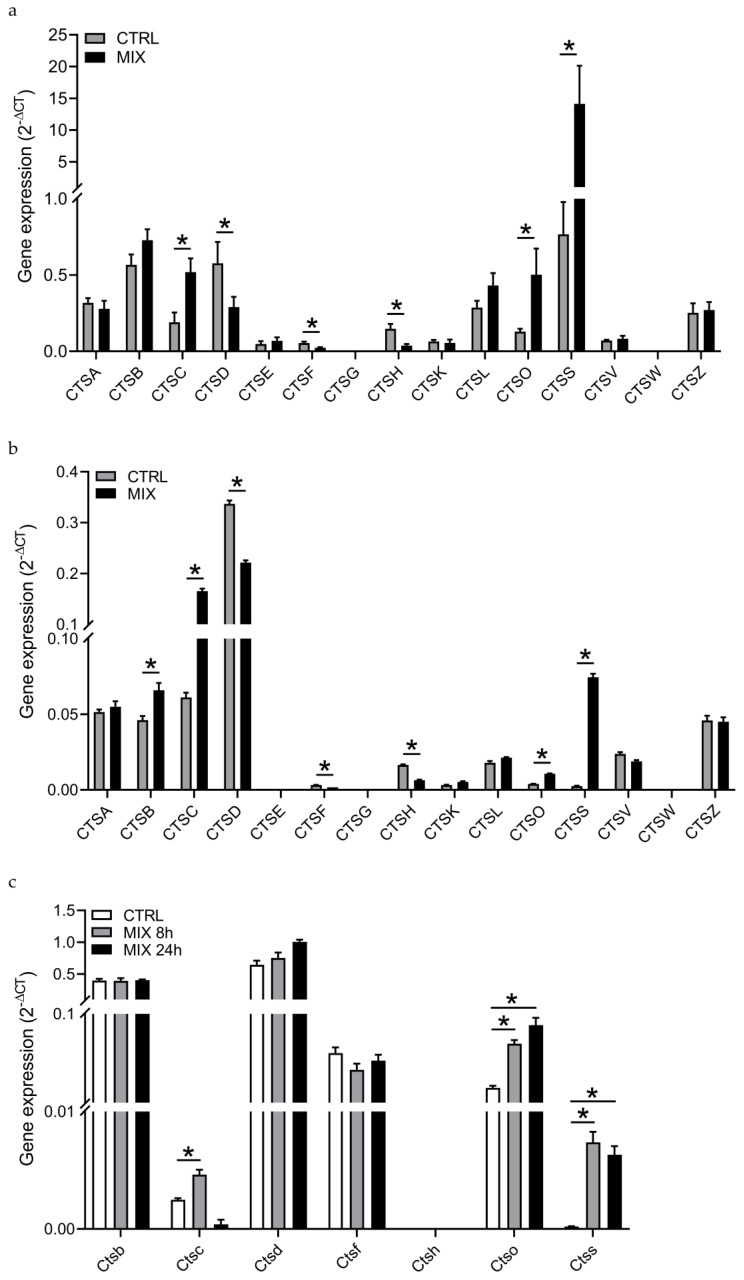
Cathepsin expression and regulation by pro-inflammatory cytokines in human pancreatic islets and β-cell models. (**a**) Gene expression of the 15 cathepsins in isolated human pancreatic islets left untreated (CTRL) or stimulated with IL-1β and IFN-γ for 24 h (MIX) as measured by real-time qPCR. The data were normalized to the geometric mean of the three housekeeping genes *ACTB*, *GAPDH* and *HPRT*. The data are presented as 2^−ΔCT^ with means ± SEM (*n* = 5). *CTSG* and *CTSW* were not detected. *: BH-adjusted *p* < 0.05. (**b**) Gene expression of the 15 cathepsins in the human 1.1B4 fusion cell line at the 0 h control condition (CTRL) and after treatment with IL-1β, IFN-γ and TNF-α for 24 h (MIX) as measured by real-time qPCR. The data were normalized to *GAPDH*. The data are presented as 2^−ΔCT^ with means ± SEM (*n* = 4). *CTSE*, *CTSG* and *CTSW* were not detected. *: Bonferroni-adjusted *p* < 0.05. (**c**) Gene expression of *Ctsb*, *Ctsc*, *Ctsd*, *Ctsf*, *Ctsh* (not expressed), *Ctso* and *Ctss* in INS-1E cells treated with IL-1β and IFN-γ for 0 (CTRL), 8 (MIX 8h) and 24 (MIX 24 h) hours as measured by real-time qPCR. The data were normalized to *Hprt*. The data are presented as 2^−ΔCT^ with means ± SEM (*n* = 4). *: Bonferroni-adjusted *p* < 0.05.

We have previously demonstrated that the T1D candidate gene *CTSH* is expressed and downregulated by pro-inflammatory cytokines in human pancreatic islets, primary rat β cells, as well as in the human 1.1B4 cell line [15,19]. To investigate if the cathepsin proteases in general are expressed and regulated by pro-inflammatory cytokines, we examined the expression of the 15 human cathepsins by real-time qPCR in isolated human pancreatic islets left untreated or exposed to IL-1β+IFN-γ for 24 h. The results showed that 13 cathepsins are expressed in human islets (Figure 1a). Cathepsin G (*CTSG*) and W (*CTSW*) were not detected. Six cathepsins were differentially expressed in human islets exposed to IL-1β+IFN-γ; cathepsin C (*CTSC*), O (*CTSO*) and S (*CTSS*) were upregulated, whereas *CTSD*, F (*CTSF*) and *CTSH* were downregulated (BH-corrected *p* < 0.05; Figure 1a). All 15 cathepsins were then examined in 1.1B4 cells, which is a recently established hybrid of a primary human β cell and the pancreatic ductal cell line PANC-1 [25]. The results showed that 12 cathepsins are expressed in 1.1B4 cells (Figure 1b). Cathepsin E (*CTSE*), *CTSG* and *CTSW* were not detected. Seven cathepsins were differentially expressed in 1.1B4 cells after 24 h of exposure to IL-1β+IFN-γ+TNF-α; *CTSB*, *CTSC*, *CTSO* and *CTSS* were upregulated, whereas cathepsin D (*CTSD*), *CTSF* and *CTSH* were downregulated (Bonferroni-adjusted *p* < 0.05; Figure 1b). The seven differentially expressed cathepsins were then examined in rat INS-1E cells after exposure to IL-1β+IFN-γ for 8 and 24 h and compared to untreated cells. Except for *Ctsh*, the selected cathepsins were expressed in INS-1E cells (Figure 1c). Three cathepsins were differentially expressed in response to cytokines; *Ctsc* was upregulated after 8 h, and *Ctso* and *Ctss* were upregulated at both 8 and 24 h, as compared to untreated INS-1E cells (Bonferroni-adjusted *p* < 0.05; Figure 1c).

Thus, in human islets, 1.1B4 cells and/or INS-1E cells, cathepsin C, D, F, H, O and S all showed to be transcriptionally regulated by pro-inflammatory cytokines known to be secreted from invading immune cells during β-cell destruction in T1D.

### 3.2. Cathepsin C Is Anti-Apoptotic in β-Cell Models

**Figure 2:**
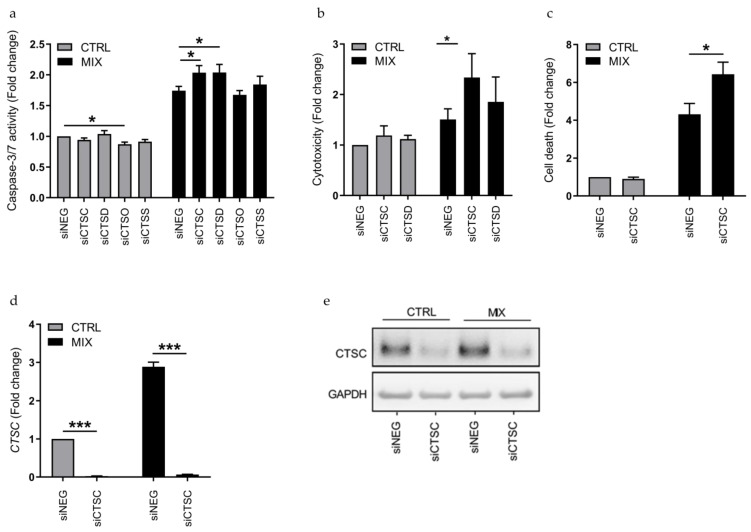
Knockdown of *CTSC* increases caspase-3/7 activity, cytotoxicity and cell death in 1.1B4 cells. (**a**) Caspase-3/7 activity in 1.1B4 cells transfected with siRNAs against *CTSC* (siCTSC), *CTSD* (siCTSD), *CTSO* (siCTSO), *CTSS* (siCTSS) or a non-targeting negative control siRNA (siNEG) and left untreated (CTRL) or exposed to IL-1β, IFN-γ and TNF-α for 24 h (MIX). Caspase-3/7 activities were normalized to total cell content. The data are presented as fold changes with means ± SEM (*n* = 6). (**b**) Cytotoxicity in 1.1B4 cells transfected with siCTSC, siCTSD or siNEG and left untreated (CTRL) or exposed to IL-1β, IFN-γ and TNF-α for 24 h (MIX). Cytotoxicity measurements were normalized to total cell content. The data are presented as fold changes with means ± SEM (*n* = 4). (**c**) Cell death in 1.1B4 cells transfected with siCTSC or siNEG and left untreated (CTRL) or exposed to IL-1β, IFN-γ and TNF-α for 24 h (MIX) as measured by the Cell Death Detection ELISA. Data were normalized to DNA content (**d**) mRNA expression of *CTSC* in 1.1B4 cells treated as in (c), analyzed by real-time qPCR and normalized to *GAPDH*. Data are presented as fold changes with means ± SEM (*n* = 4). (**e**) Protein level of CTSC in 1.1B4 cells treated as in (**c**), analyzed by immunoblotting with GAPDH as loading control. *: *p* < 0.05; **: *p* < 0.01; ***: *p* < 0.001.

With the aim of investigating if the cytokine-regulated cathepsins are modulators of β-cell apoptosis, we examined if individual KD of the cathepsins using RNAi affected cytokine-induced caspase-3/7 activity in 1.1B4 cells. Since we previously established the involvement of cathepsin H in cytokine-induced β-cell apoptosis in INS-1 and 1.1B4 cells [15,19], we excluded it from these experiments. siRNAs were used to KD the expression of *CTSC*, *CTSD*, *CTSO* and *CTSS* in 1.1B4 cells, since these cathepsins were differentially expressed and their expression levels had sufficient silencing potential. KD of the individual cathepsins using gene-specific siRNA pools was compared to control cells transfected with a non-targeting negative control siRNA pool (siNEG). Interestingly, KD of *CTSC* and *CTSD* caused a significant increase in cytokine-induced caspase-3/7 activity, whereas KD of *CTSO* modestly, but significantly, decreased basal caspase-3/7 activity (*p* < 0.05; Figure 2a). KD of *CTSS* neither affected basal nor cytokine-induced caspase-3/7 activity in 1.1B4 cells (Figure 2a). The effects of *CTSC* and *CTSD* KD on caspase-3/7 activity were further investigated by cytotoxicity measurements. KD of *CTSC* led to increased cytokine-induced cytoxicity in the 1.1B4 cells, whereas KD of *CTSD* did not significantly affect cytokine-induced cytotoxicity (Figure 2b). The anti-apoptotic effect of cathepsin C was further verified by measurement of cytoplasmic nucleosomes indicative of apoptotic cell death, where KD of *CTSC* increased cell death (*p* < 0.05; Figure 2c). Efficient siRNA-mediated KD of *CTSC* in 1.1B4 cells was verified at mRNA level by real-time qPCR (Figure 2d) and at protein level by immunoblotting (Figure 2e). Real-time qPCR showed a KD efficacy of >95%, as compared to the *CTSC* mRNA expression in control cells transfected with siNEG (Figure 2d).

**Figure 3:**
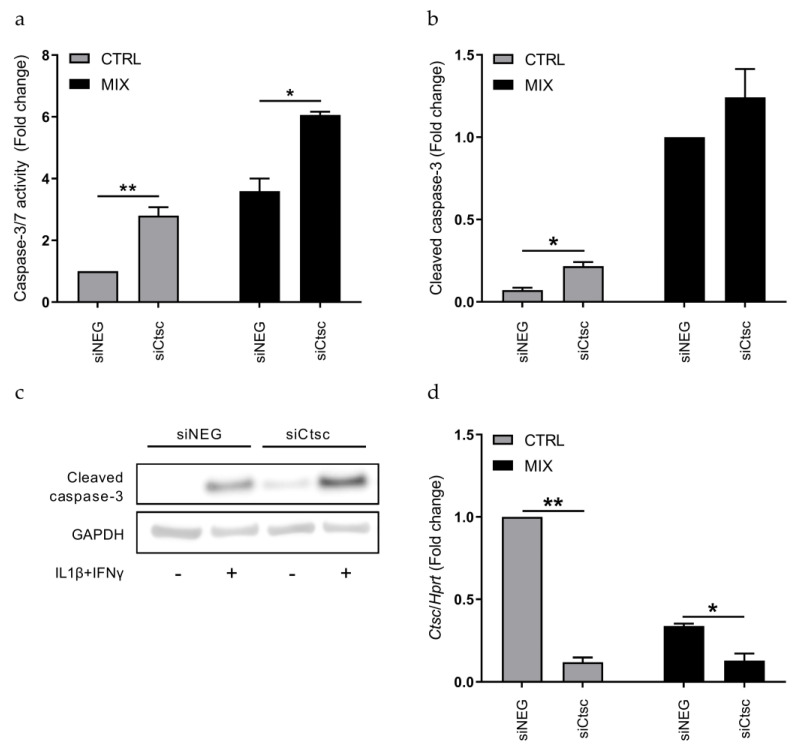
Knockdown of *Ctsc* increases caspase-3/7 activity in INS-1E cells. INS-1E cells were transfected with siRNAs against *Ctsc* (siCtsc) or a non-targeting negative control siRNA (siNEG) and then left untreated (CTRL) or exposed to IL-1β and IFN-γ for 24 h (MIX). (**a**) Caspase-3/7 activity was normalized to total cell content, and data are presented as fold changes with means ± SEM (*n* = 4). (**b**) Cleaved caspase-3 protein level as analyzed by immunoblotting. Gapdh was used as loading control. Data are presented as fold changes with means ± SEM (*n* = 4). (**c**) Visualized protein bands of cleaved caspase-3 (~17–19 kDa) and Gapdh (~40 kDa), as presented in (b). The blot is representative of 4 blots. (**d**) *Ctsc* mRNA expression measured by real-time qPCR and normalized against *Hprt*. Data are presented as fold changes with means ± SEM (*n* = 4). *: *p* < 0.05; **: *p* < 0.01.

We then investigated if KD of *Ctsc* also affected cytokine-induced apoptosis in the rat INS-1E β-cell line. Using the caspase-3/7 activity assay, we observed that KD of *Ctsc* caused a significant increase in both basal and cytokine-induced caspase-3/7 activity, as compared to siNEG control cells (*p* < 0.05; Figure 3a). We also analyzed the level of cleaved caspase-3 protein by immunoblotting and found that KD of *Ctsc* led to a 3-fold increase at baseline, compared to siNEG control cells (*p* < 0.05; Figure 3b,c). Efficient siRNA-mediated KD of *Ctsc* in INS-1E cells was verified by real-time qPCR and showed that the *Ctsc*-specific siRNA pool decreased *Ctsc* mRNA expression by ~75% as compared to siNEG control cells (*p* < 0.05; Figure 3d).

### 3.3. Cathepsin C Modulates MAPK Signaling

To identify putative mechanisms of action underlying cathepsin C-regulated cell death, we examined some of the well-known signaling factors responsible for cytokine-mediated β-cell apoptosis [2,4,28].

**Figure 4:**
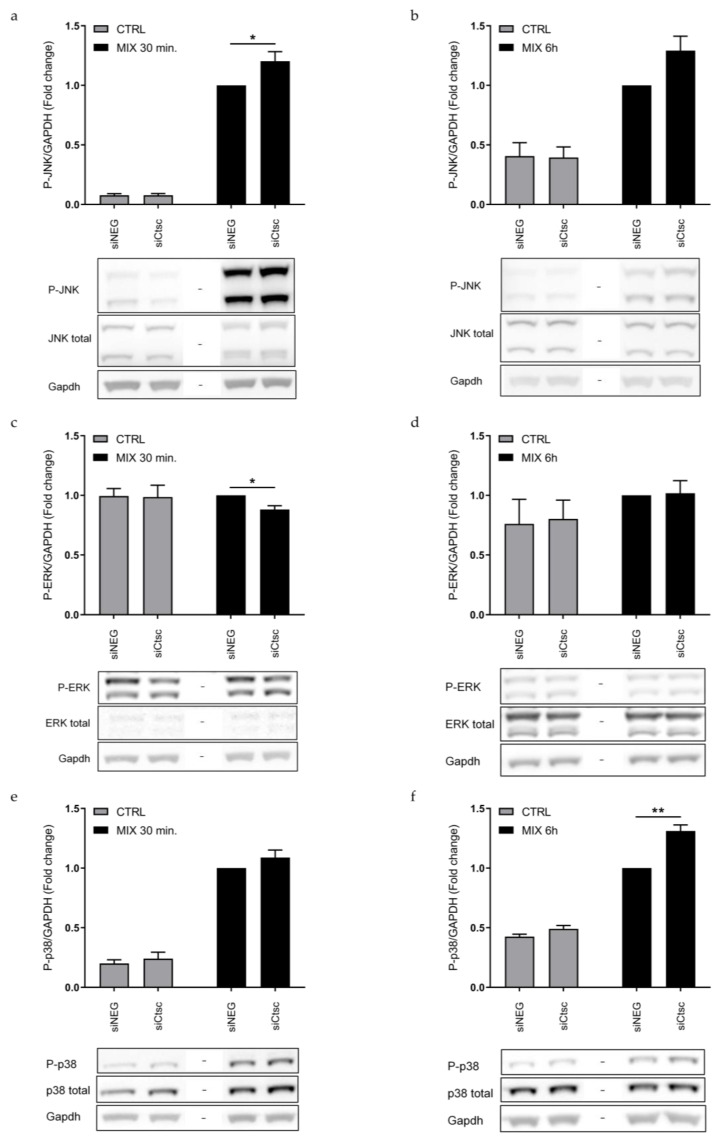
Knockdown of *Ctsc* regulates MAPK signaling. INS-1E cells were transfected with siRNA against *Ctsc* (siCtsc) or a non-targeting negative control siRNA (siNEG) and were then left untreated (CTRL) or exposed to IL-1β and IFN-γ (MIX) for 30 min or 6 h. Immunoblotting of phosphorylated JNK after (**a**) 30 min and (**b**) 6 h, phosphorylated ERK after (**c**) 30 min and (**d**) 6 h, and phosphorylated p38 after (**e**) 30 min and (**f**) 6 h. Gapdh was used as loading control. Data are presented as fold changes with mean ± SEM (*n* = 4–7). *: *p* < 0.05; **: *p* < 0.01.

Initially, we investigated if KD of *Ctsc* affected cytokine signaling through the mitogen-activated protein kinases (MAPK)s: JNK, p38 and ERK, in the INS-1E cells. Interestingly, KD of *Ctsc* led to an increase in the levels of phosphorylated (activated) JNK1/2 after 30 min of cytokine exposure and phosphorylated p38 after 6 h of cytokine exposure, as compared to siNEG control cells (*p* < 0.05; Figure 4). Additionally, KD of *Ctsc* modestly reduced the phosphorylated level of ERK1/2 after 30 min of cytokine exposure, compared to siNEG control cells (Figure 4).

We also investigated if cathepsin C regulates cytokine-induced nitric oxide (NO) production by iNOS which is a critical mediator of ER stress [2]. We observed no differences in cytokine-induced iNOS protein and mRNA levels nor NO production upon *Ctsc* KD (Supplementary Figure S1). ER stress-related apoptosis signaling was further assessed by analyses of BiP, cytochrome C and phosphorylated IRE1a (Supplementary Figure S2). However, no changes were found upon *Ctsc* KD.

### 3.4. Cathepsin C Regulates CXCL10 Expression and Secretion

**Figure 5:**
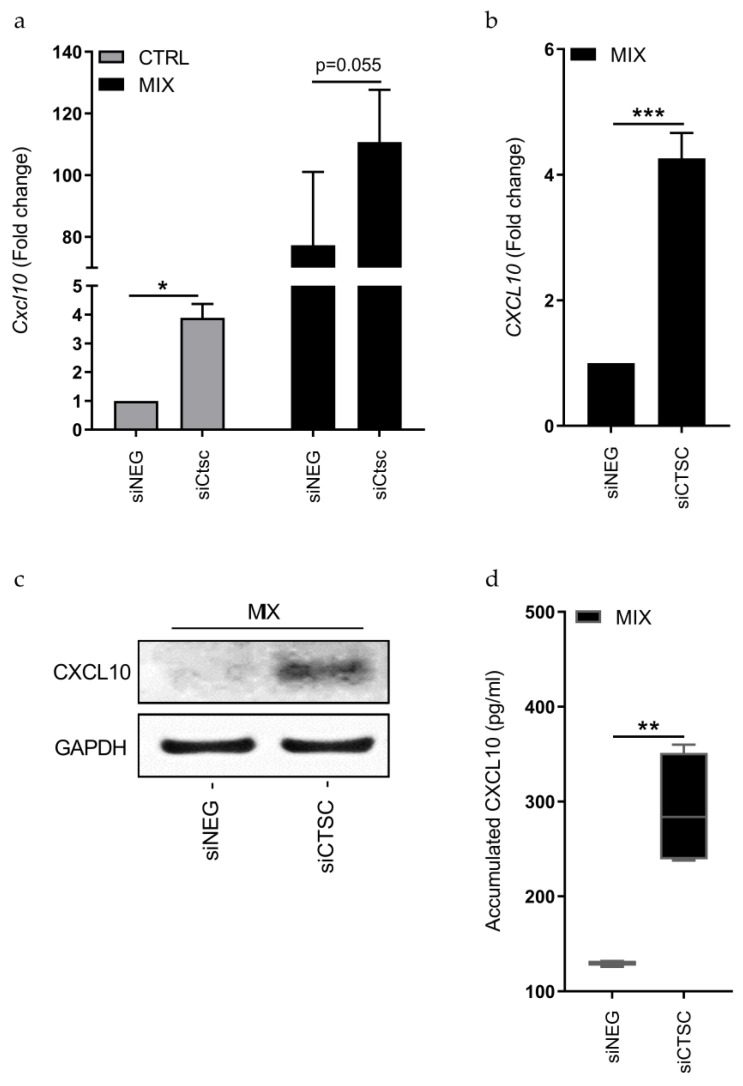
Knockdown of *CTSC* increases CXCL10 expression and secretion. (**a**) INS-1E cells were transfected with a siRNA pool against *Ctsc* (siCtsc) or a non-targeting negative control siRNA pool (siNEG) and left untreated (CTRL) or exposed to IL-1β and IFN-γ for 24 h (MIX). *Cxcl10* mRNA expression was analyzed using real-time qPCR with *Hprt* as housekeeping gene. (**b**) 1.1B4 cells were transfected with a siRNA pool against *CTSC* (siCTSC) or a non-targeting negative control siRNA pool (siNEG) and left untreated (CTRL) or exposed to IL-1β and IFN-γ for 24 h (MIX). *CXCL10* mRNA expression was analyzed using real-time qPCR with *GAPDH* as housekeeping gene. (**c**) Protein level of CXCL10 in 1.1B4 cells treated as in (**b**) with GAPDH as loading control. (**d**) Accumulated CXCL10 in the culture media from 1.1B4 cells treated as in (**b**). Graphs are presented as fold change with mean and SEM or pg/mL with median and 5/95 percentiles (*n* = 4), (**a**) Bonferroni-adjusted * *p* < 0.05, (**b**,**d**) ** *p* < 0.01, *** *p* < 0.001.

Using real-time qPCR, we next investigated potentially affected downstream genes in the cytokine signaling pathways (*Jun*, *Fos, Myc, Bim*/*Bcl2l11, Ddit3*/*Chop,* and *Cxcl10*) in INS-1E cells at baseline and in response to 6 and 24 h of cytokine exposure (Supplementary Figure S3). We found only moderate changes in cytokine-regulated expression of these genes after KD of *Ctsc* as compared to siNEG control cells (Supplementary Figure S3). However, at baseline, *Cxcl10* was significantly upregulated upon *Ctsc* KD (*p* < 0.01; Figure 5a). Additionally, after 24 h of cytokine exposure there was a strong trend towards upregulation of *Cxcl10* upon *Ctsc* KD (*p* = 0.055, Figure 5a).

In 1.1B4 cells, there were no basal expression of *CXCL10*, however, in response to 24 h of cytokine exposure, the *CXCL10* expression was 4.3-fold higher upon *CTSC* KD, as compared to the siNEG-transfected cells (*p* < 0.001; Figure 5b). Immunoblotting confirmed increased CXCL10 protein after 24 h of cytokine exposure in response to *CTSC* KD (Figure 5c). Finally, we investigated if the observed effects of *CTSC* KD on CXCL10 expression were accompanied by increased CXCL10 secretion to the culture medium. Using Luminex bead-based multiplexing immunoassays, we observed a 2.2-fold increase in accumulated CXCL10 in the cell culture medium of cells with *CTSC* KD, as compared to siNEG transfected cells after 24 h of cytokine exposure *(p* < 0.01, Figure 5d).

## 4. Discussion

In this study, we report that pro-inflammatory cytokines modulate the expression of several cathepsin proteases in human islets and β-cell models. We further report that KD of *CTSC* caused increased apoptotic cell death, indicative of an anti-apoptotic function of this cathepsin in β cells. Signal transduction studies suggested that cathepsin C regulates pro-apoptotic signal transduction via the JNK and p38 MAPKs. Further, our data suggest that cathepsin C regulates the expression and secretion of the chemokine CXCL10.

Noteworthy, only around 20% of β-cell-encoded genes are regulated in response to pro-inflammatory cytokines [29]. We found that approximately 50% of the cathepsins were transcriptionally regulated by cytokines in human islets and 1.1B4 cells. This highlights the significance of the number of differentially expressed cathepsin members observed by the present study and proposes a role for them in β-cell signaling and T1D pathogenesis. Already, several of the cathepsins have been studied in insulitis and immune-mediated β-cell death by others, supporting that these proteases likely play critical roles in T1D. Using cathepsin knockout mice or cathepsin inhibitors, studies have found that cathepsin B, G, L and S are important for the onset of insulitis and autoimmune diabetes in nonobese diabetic (NOD) mice [30,31,32,33]. Cathepsin C, W and S are found at sites of immune cell infiltration in pancreatic islet samples from NOD mice and human donors with T1D, suggesting that they are secreted during early stages of insulitis causing degradation of extracellular matrix proteins [34]. Furthermore, the cathepsins have been investigated for their involvement in processing of diabetogenic epitopes [33,35]. Zou et al. found that cathepsins derived from B cells and myeloid dendritic cells cleave proinsulin, one of the main autoantigens in T1D [36]. Proinsulin processing by cathepsin G was found by the study to be crucial for the generation of proinsulin-reactive T cells [33]. Interestingly, the expression and activity of cathepsin G is elevated in peripheral blood mononuclear cells (PBMC) from patients with T1D compared to healthy controls, as well as in CD4+ T cells from diabetic NOD mice [33,36]. Also, the level of cathepsin S has been shown to be increased in serum from children with T1D as compared to healthy control subjects [37]. These studies suggest a potential of several of the cathepsin proteases as therapeutic targets in T1D [30,32,33,34]. However, aside from their distinct functions and individual disease associations, compensatory redundancy between the cathepsin family members have previously been found [38,39,40,41]. Hence, the causative role of the cathepsins may lie in an overall dysregulation of the entire cathepsin expression and/or activity profile. To understand this intricate balancing of the cathepsins and the consequence of their dysregulation, further studies of their interaction networks and signaling pathways are warranted.

Previously, we demonstrated that cathepsin H is a key player in β-cell survival. Specifically, we showed that overexpression of *CTSH* protected against cytokine-induced apoptosis by reducing signaling via the JNK and p38 MAPKs in insulin-producing cells [15]. In the present study, we emphasize the putative roles of other cathepsin proteases in detrimental cytokine-mediated β-cell signaling, showing that five other cathepsins (C, D, F, O and S), besides *CTSH*, are significantly regulated by pro-inflammatory cytokines and that cathepsin C additionally regulates cytokine-induced apoptosis in the β cells.

Cathepsin C, also known as dipeptidyl peptidase-I (DPP-I), is an exo-cysteine protease, known for its roles in zymogen activation in secretory granules of immune cells [42]. To our knowledge, cathepsin C has not previously been directly linked to β-cell apoptosis, however, it has been identified as a cell death regulator in other cell types. Khaket et al. reported that KD of cathepsin C increased curcumin-induced apoptosis, and that *CTSC* KD and curcumin treatment upregulate ER stress and autophagic dysfunction in colorectal cancer cells [43]. Others have identified cathepsin C as an important regulator in pyroptosis and lysosome-mediated cell death in cathepsin C deficient mouse splenocytes [44,45].

In diabetes, cathepsin C has only been studied for its differential expression and activity in immune cells [46,47]. In a small study, Orban et al. detected a lower gene expression level of *CTSC* in CD4+ T cells derived from newly diagnosed individuals with T1D, as compared to healthy controls and individuals with T2D [46]. Another study found the enzymatic activities of cathepsin C, B and L to be increased in leukocytes and monocytes from individuals with T2D, as compared to healthy controls [47]. Otherwise, cathepsin C has been studied for its role in Papillon-Lefevre and Haim-Munk syndrome, where a loss-of-function mutation in the *CTSC* gene causes inactivation of neutrophil serine peptidases, loss of neutrophil extracellular trap production and defective neutrophil chemotaxis [48].

The role of cathepsin C in modulating chemoattraction and immune regulation was also previously studied [49,50,51]. Zhao et al. recently identified cathepsin C as a regulator of several chemokines and cytokines in overexpression and KD mice studies, showing that cathepsin C aggravates neuroinflammation by promoting glial cell and neuron chemokine production at brain lesion sites [51]. In another study, investigating the functional role of secretory cathepsin C in breast cancer lung metastasis, cathepsin C was reported to activate neutrophil membrane-bound proteinase-3 (PR3), upregulate IL-1β secretion, and activate p38 and nuclear factor (NF)-κB signaling, thus leading to enhanced neutrophil recruitment [49]. Correspondingly, Alam et al. identified cathepsin C as a regulator of the p38/NF-κB signaling pathway in mouse peritoneal macrophages and a macrophage cell line upon treatment with cathepsin C [50]. Furthermore, cathepsin C treatment led to the upregulation of cytokine gene expression, facilitating macrophages toward M1 differentiation [50]. In the present study, we similarly identify cathepsin C as a regulator of the MAPKs p38 and JNK.

Our results endorse the role of cathepsin C in chemotaxis by affecting the expression and secretion of CXCL10. CXCL10 is thought to be a key chemoattractant in diabetes pathogenesis and has been found elevated at early stages of T1D in rodent and human studies [52,53,54], including in serum from individuals with newly diagnosed T1D [55,56]. Furthermore, Yoshimatsu et al. suggested that CXCL10 is regulated by stress-induced MAPK signaling in β cells in response to IL-1β exposure [54]. Inhibition of JNK and p38 reduced CXCL10 expression and secretion upon treatment with IL-1β and high glucose in human pancreatic islets [54]. This proposes a dual role of the MAPK and chemokine signaling in islet inflammation, which encourages future exploration into the MAPK-CXCL10 relationship, and its regulation by cathepsin C.

Thus, cathepsin C appears to participate in the inflammatory β-cell response both by regulating intracellular apoptosis signaling pathways and extracellular chemokine-mediated crosstalk. The main limitation of the present study is that only a subset of genes and proteins known to be involved in β-cell apoptosis has been investigated upon cathepsin C KD. To fully understand the molecular mechanism(s) through which cathepsin C regulates β-cell apoptosis, exploratory studies should focus on identifying the proteins cleaved by cathepsin C. It would therefore be highly relevant to investigate changes in the proteome signature in response to cathepsin C KD. Additionally, the results of the present study should be substantiated by studies investigating the effect of inhibiting cathepsin C activity, e.g., through treatment with pharmacological inhibitors. Furthermore, as the transcriptional changes occurring within the targeted β cells can act both as contributory and counteracting factors in the insulitis and immune cell cross-talk [29], perhaps the cytokine-induced upregulation of cathepsin C observed in the present study represents a defense mechanism against the immune attack. Overexpression studies investigating this are needed to fully understand the implication of cathepsin C in β-cell survival.

In conclusion, cathepsin C, like previously observed for cathepsin H, contributes to immune-mediated destruction of the β cells, calling for further investigations into the cathepsin protease family and their roles in β-cell signaling and T1D.

## Supplementary Materials

The following are available online at https://www.mdpi.com/article/10.3390/genes12111694/s1, Figure S1: Effect of *Ctsc* KD on iNOS, NO production and *Nos2* mRNA expression, Figure S2: Effect of *Ctsc* KD on BiP, cytochrome c and p-IREa, Figure S3: Effect of *Ctsc* KD on downstream MAPK signaling gene expression, Figure S4: Whole western blots of CTSC and CXCL10 upon *CTSC* KD in 1.1B4 cells, Figure S5: Whole western blots of cleaved caspase-3, the MAPKs and iNOS upon *Ctsc* KD in INS-1E cells, Table S1: Pancreatic islet donor information.
