# Supplementary material for: Cathepsin C Regulates Cytokine-Induced Apoptosis in β-Cell Model Systems
Source: Genes (Basel). 2021 Oct 25;12(11):1694. doi: 10.3390/genes12111694 (PMC8622156; doi:10.3390/genes12111694)
Supplement: Supplementary file 1 [file genes-12-01694-s001.zip › Supplementary Figure S1, S2, S3.pdf]

Supplementary

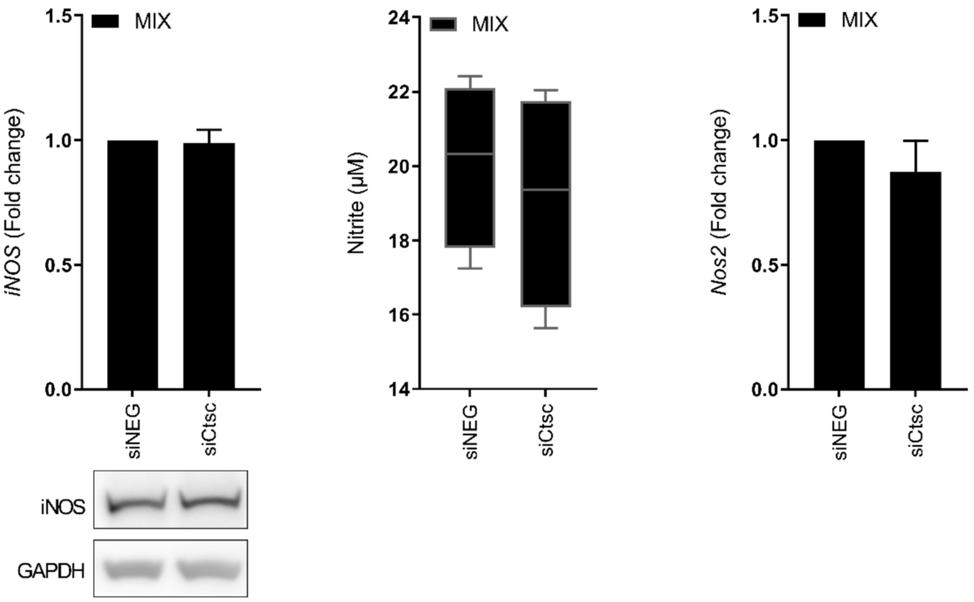

(**a**) (**b**) (**c**)

Supplementary Figure S1. Effect of *Ctsc* KD on iNOS, NO production and *Nos2* mRNA expression. INS-1E cells were transfected with a siRNA pool against *Ctsc* (siCtsc) or a non-targeting negative control siRNA pool (siNEG) and then exposed to pro-inflammatory cytokines IL-1β and IFN-γ (MIX). (**a**) iNOS protein was examined by immunoblotting after 6 h of cytokine exposure. *GAPDH* was used as loading control. Data are presented as fold change with mean and SEM (n=4). (**b**) NO production was assessed using the Griess Reagent System by measuring the accumulated nitrite in the cell culture medium after 24 h of cytokine exposure. Data are presented as (µM) with min., first quantile (25 %), median, third quantile (75 %) and max., (n=4). (**c**) The mRNA expression of iNOS (*Nos2*) was investigated by real-time qPCR after 24 h of cytokine exposure. Data were normalized to *Hprt* and presented as fold change with mean and SEM, (n=4).

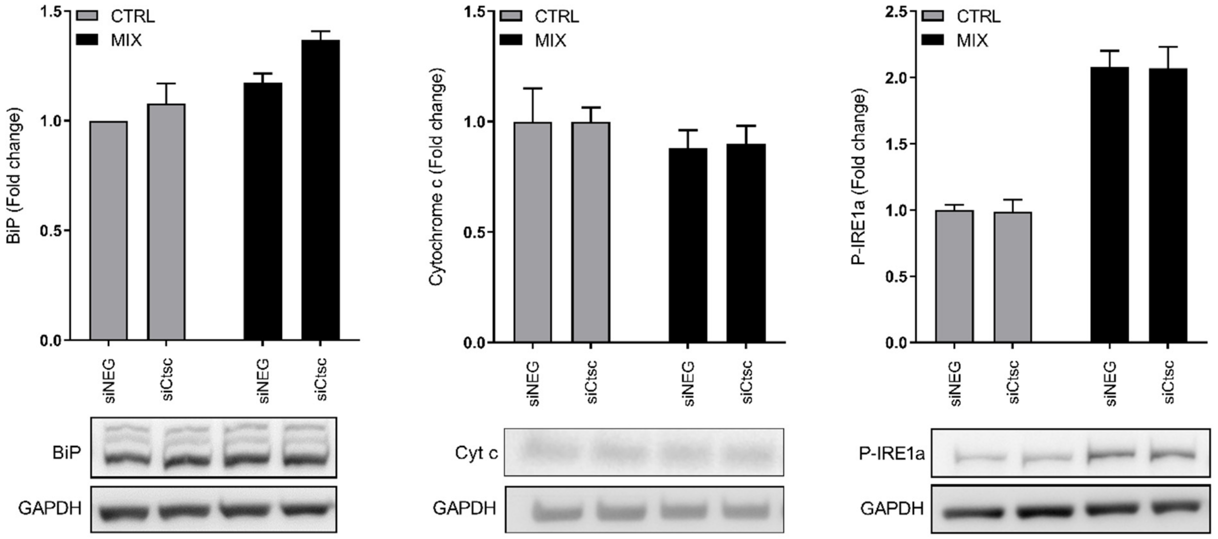

(**a**) (**b**) (**c**)

Supplementary Figure S2. Effect of *Ctsc* KD on BiP, cytochrome c and p-IREa. INS-1E cells were transfected with a siRNA pool against *Ctsc* (siCtsc) or a non-targeting negative control siRNA pool (siNEG) and then exposed to pro-inflammatory cytokines (IL-1β and IFN-γ). (**a**) BiP, (**b**) cytochrome c, and (**c**) p-IRE1a were evaluated at the protein level by immunoblotting at baseline and after 6 h of cytokine exposure. *GAPDH* was used as loading control. Data are presented as fold change with mean and SEM (n=4).

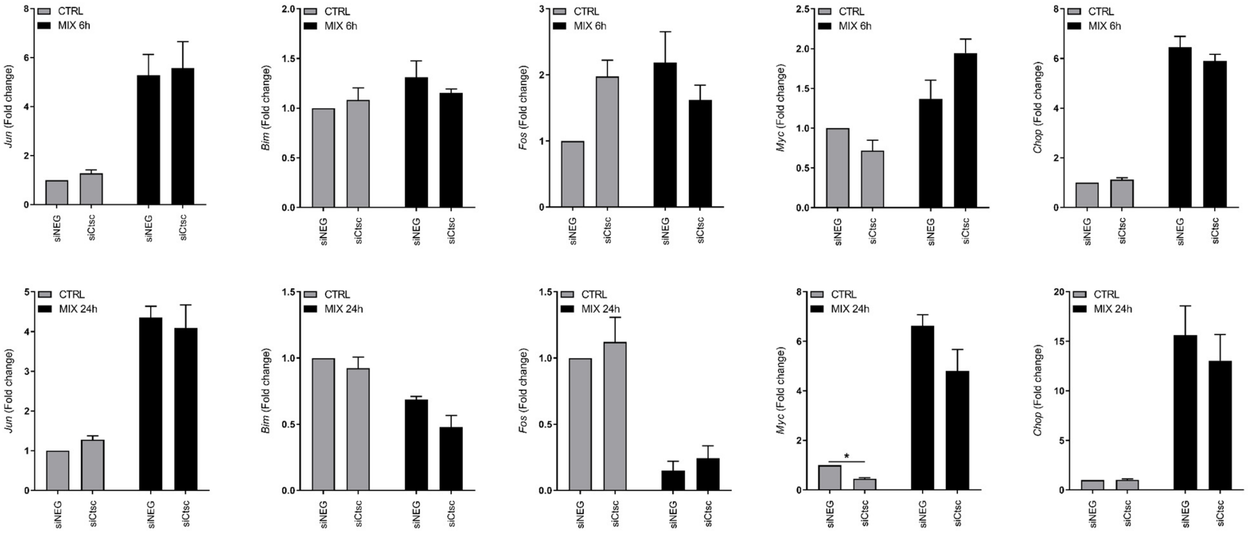

(**a**)

(**b**)

Supplementary Figure S3. Effect of *Ctsc* KD on downstream MAPK signaling gene expression. INS-1E cells were transfected with a siRNA pool against *Ctsc* (siCtsc) or a non-targeting negative control siRNA pool (siNEG) and then exposed to pro-inflammatory cytokines (IL-1β and IFN-γ). The mRNA expression of *Jun*, *Bim*/*Bcl2l11*, *Fos*, *Myc*, and *Ddit3*/*Chop*, were analyzed by real-time qPCR after (**a**) 6 and (**b**) 24 h of cytokine exposure. Data were normalized to *Hprt1* and presented as fold change with mean and SEM, (n=4 (**a**) and n=5 (**b**)). Bonferroni-adjusted p<0.05.
