# Supplementary material for: Cathepsin C Regulates Cytokine-Induced Apoptosis in β-Cell Model Systems
Source: Genes (Basel). 2021 Oct 25;12(11):1694. doi: 10.3390/genes12111694 (PMC8622156; doi:10.3390/genes12111694)
Supplement: Supplementary file 1 [file genes-12-01694-s001.zip › Supplementary Figure S4 and S5.pdf]

Supplementary Figures – whole western blots

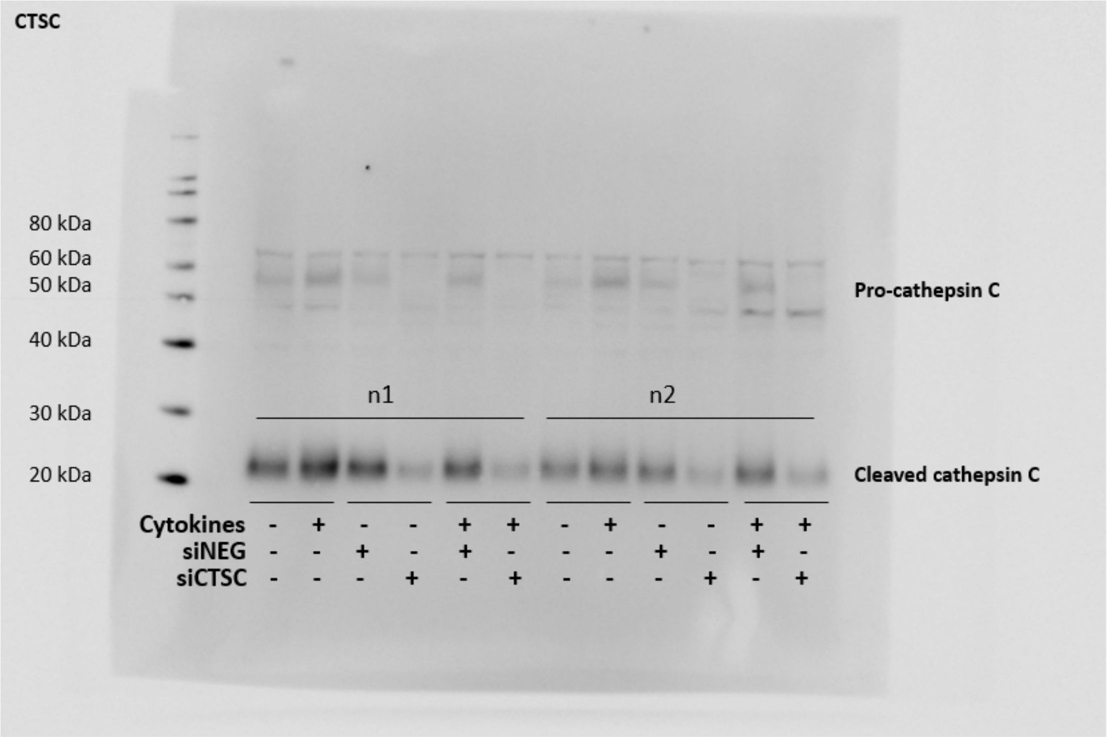

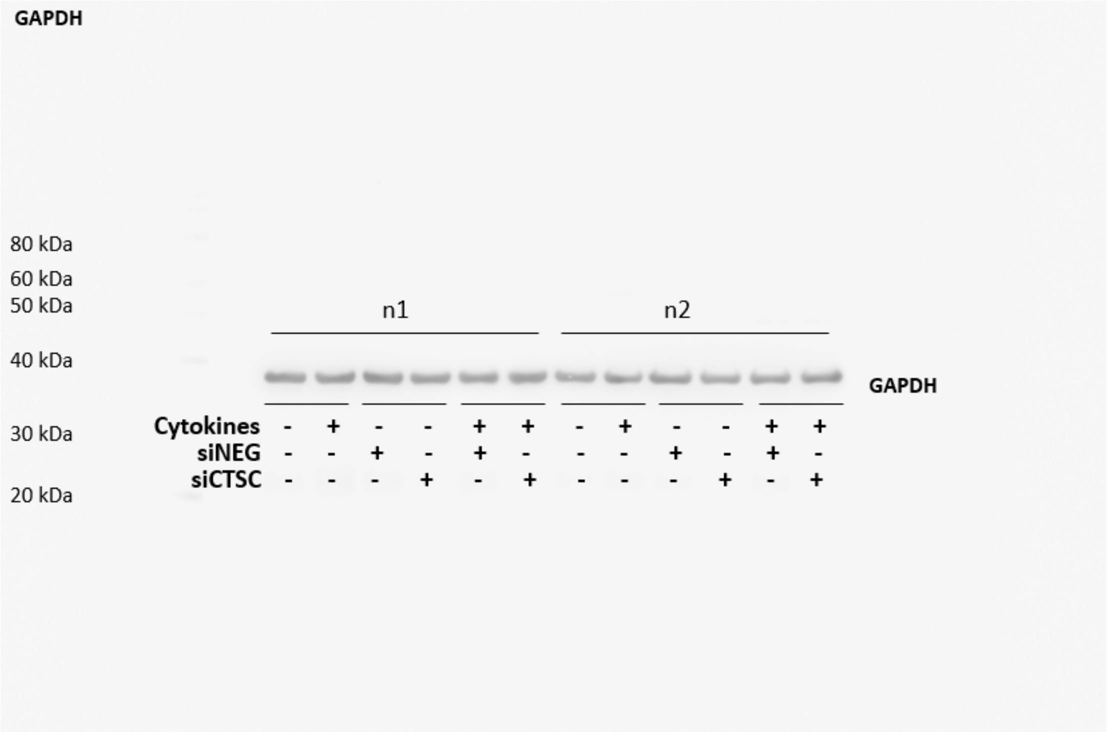

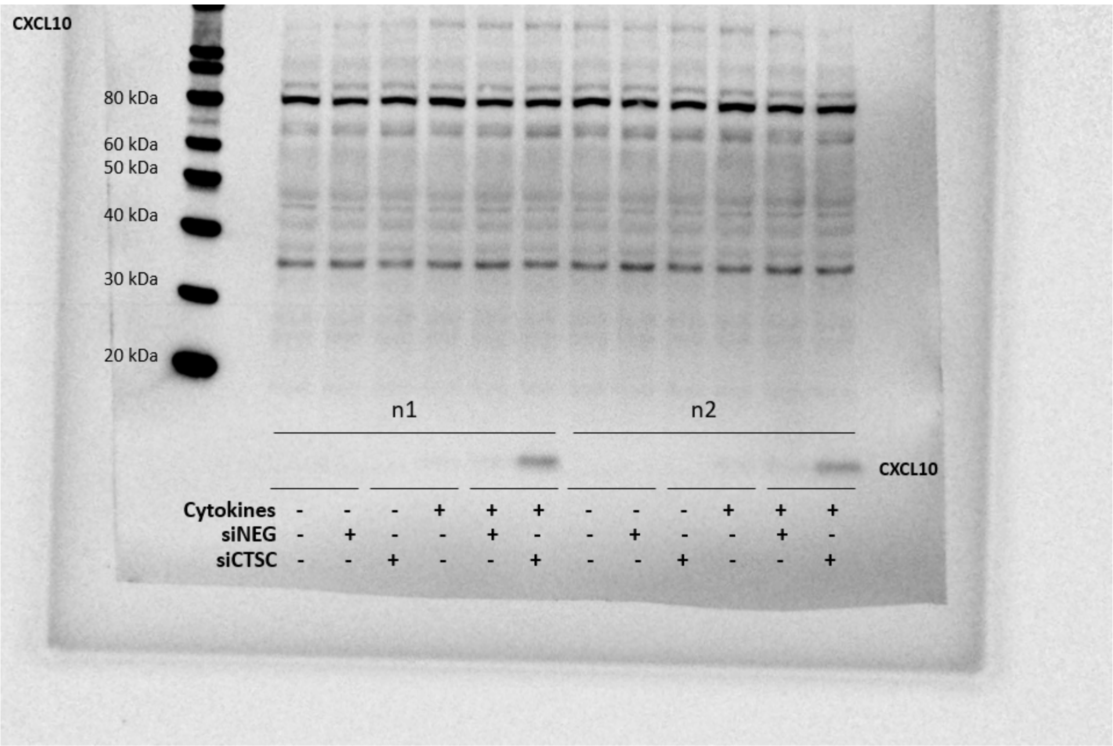

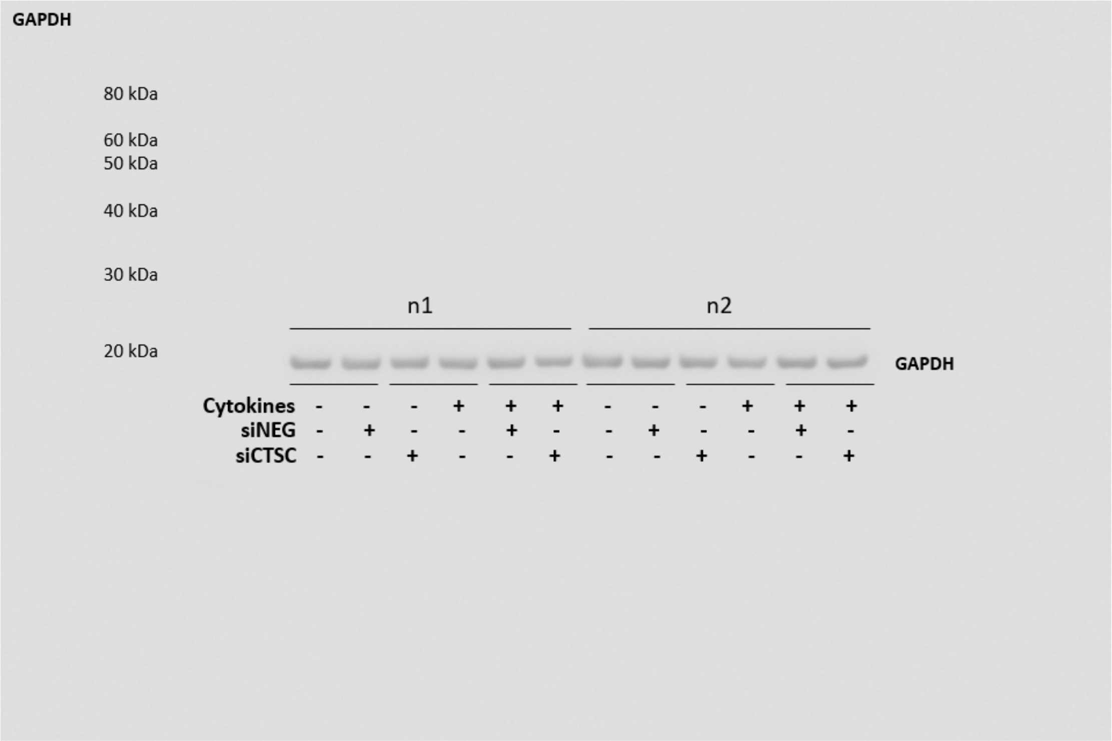

Supplementary Figure S4. Whole western blots of CTSC and CXCL10 upon *CTSC* KD in 1.1B4 cells. Immunoblotting of CTSC and CXCL10 in 1.1B4 cells transfected with siRNA against *CTSC* or a non-targeting negative control siRNA, untreated or exposed to IL-1β, IFN-γ and TNF-α for 24 h. GAPDH was used as loading control.

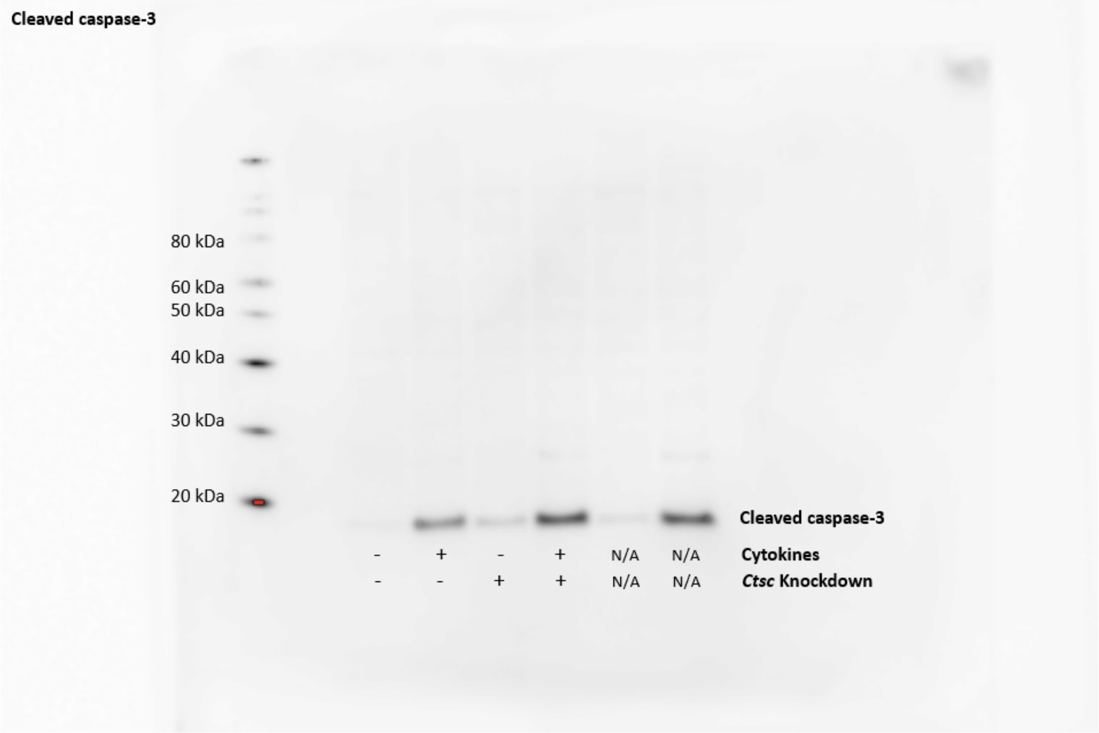
Cleaved caspase-3
80 kDa
60 kDa
50 kDa
40 kDa
30 kDa
20 kDa
Cleaved caspase-3
- + - + N/A N/A Cytokines
- - + + N/A N/A *Ctsc* Knockdown

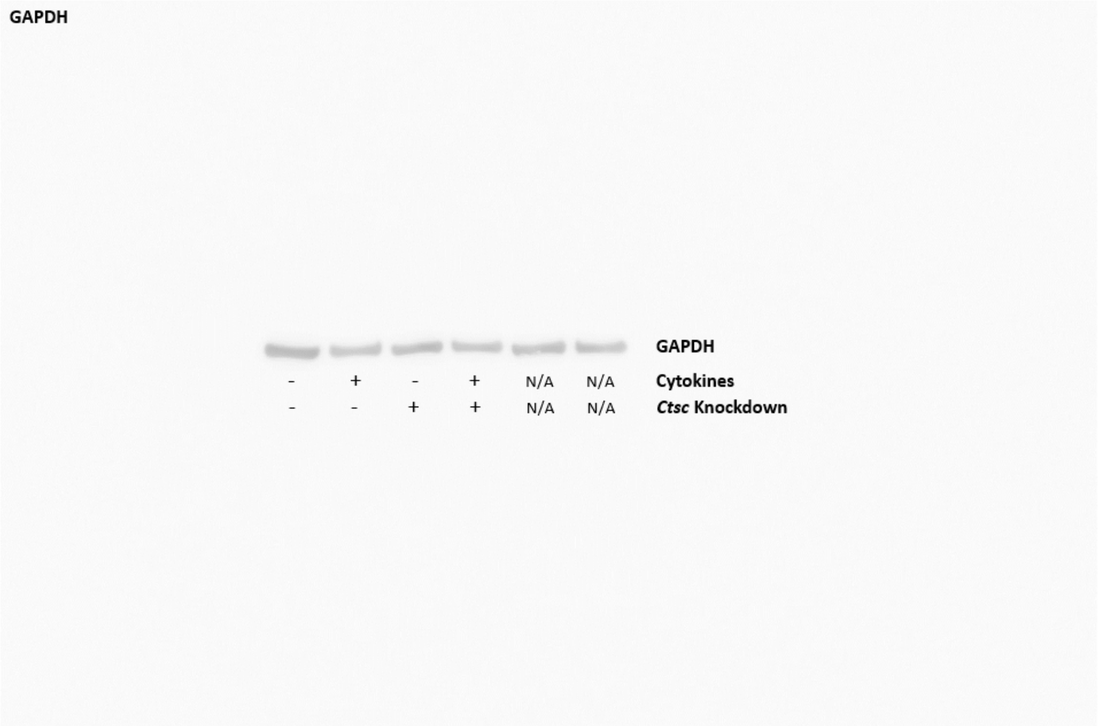
GAPDH
GAPDH
- + - + N/A N/A Cytokines
- - + + N/A N/A *Ctsc* Knockdown

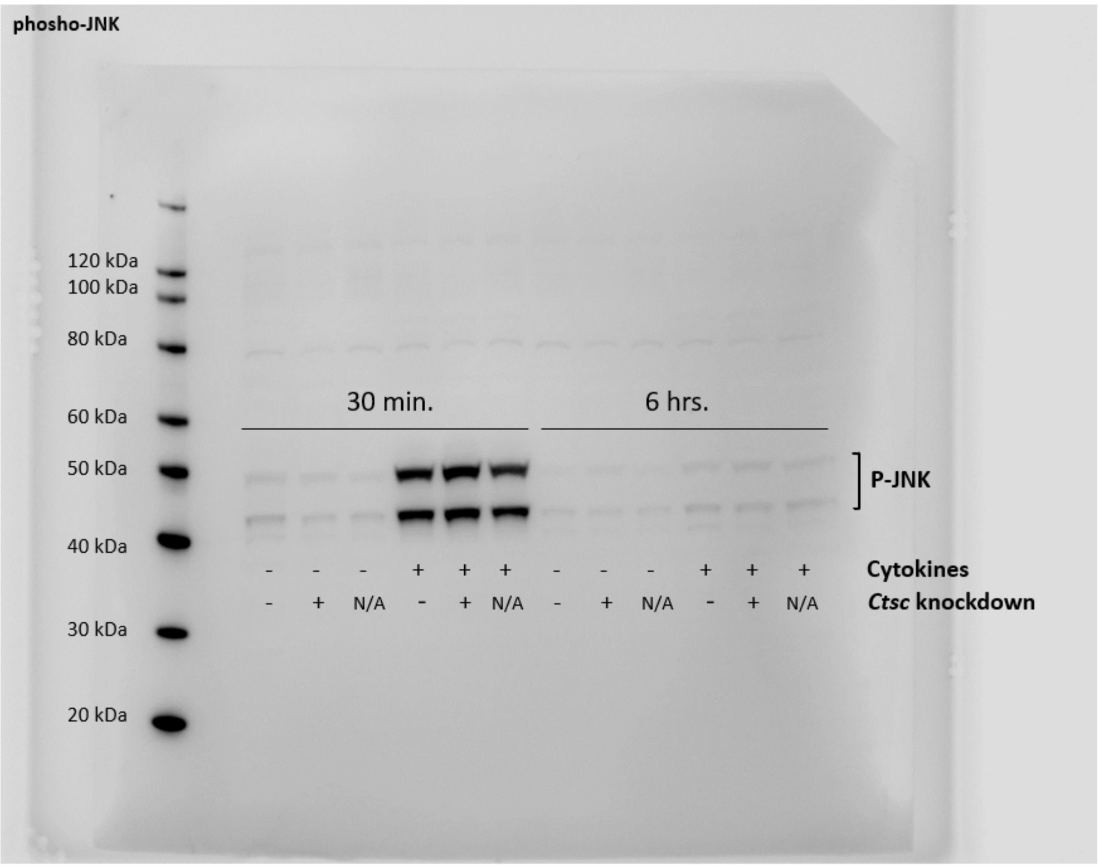
phosho-JNK
120 kDa
100 kDa
80 kDa
60 kDa
50 kDa
40 kDa
30 kDa
20 kDa
30 min.
6 hrs.
P-JNK
- - - + + + - - - + + +
Cytokines
- + N/A - + N/A - + N/A - + N/A
Ctsc knockdown

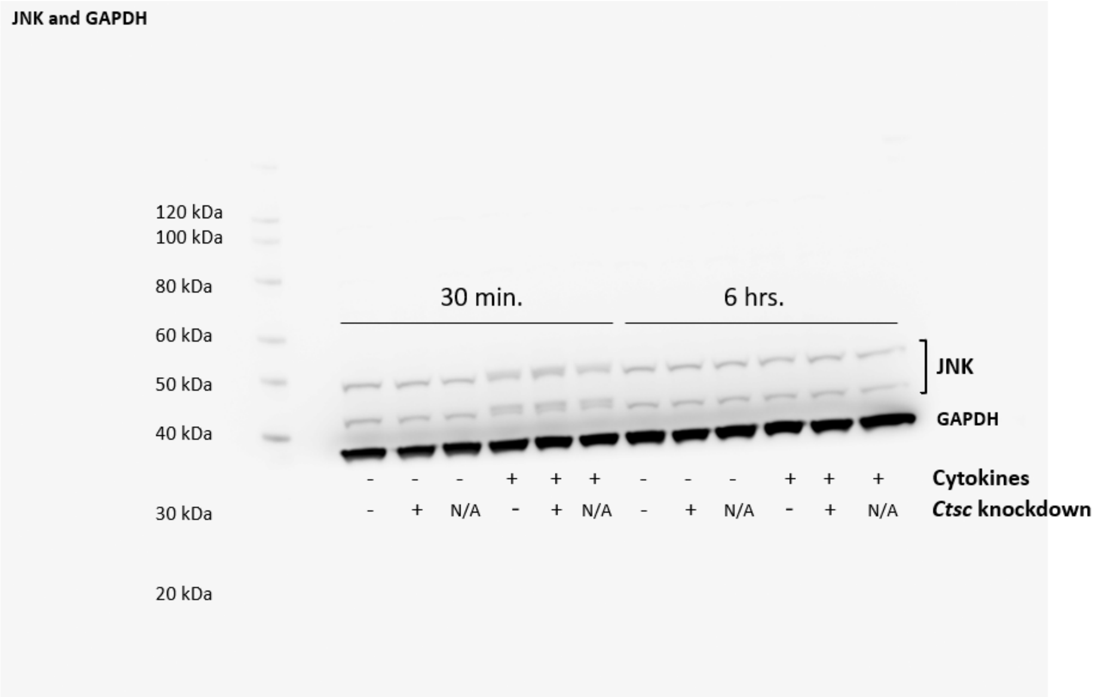
JNK and GAPDH
120 kDa
100 kDa
80 kDa
60 kDa
50 kDa
40 kDa
30 kDa
20 kDa
30 min.
6 hrs.
JNK
GAPDH
- - - + + + - - - + + +
Cytokines
- + N/A - + N/A - + N/A - + N/A
Ctsc knockdown

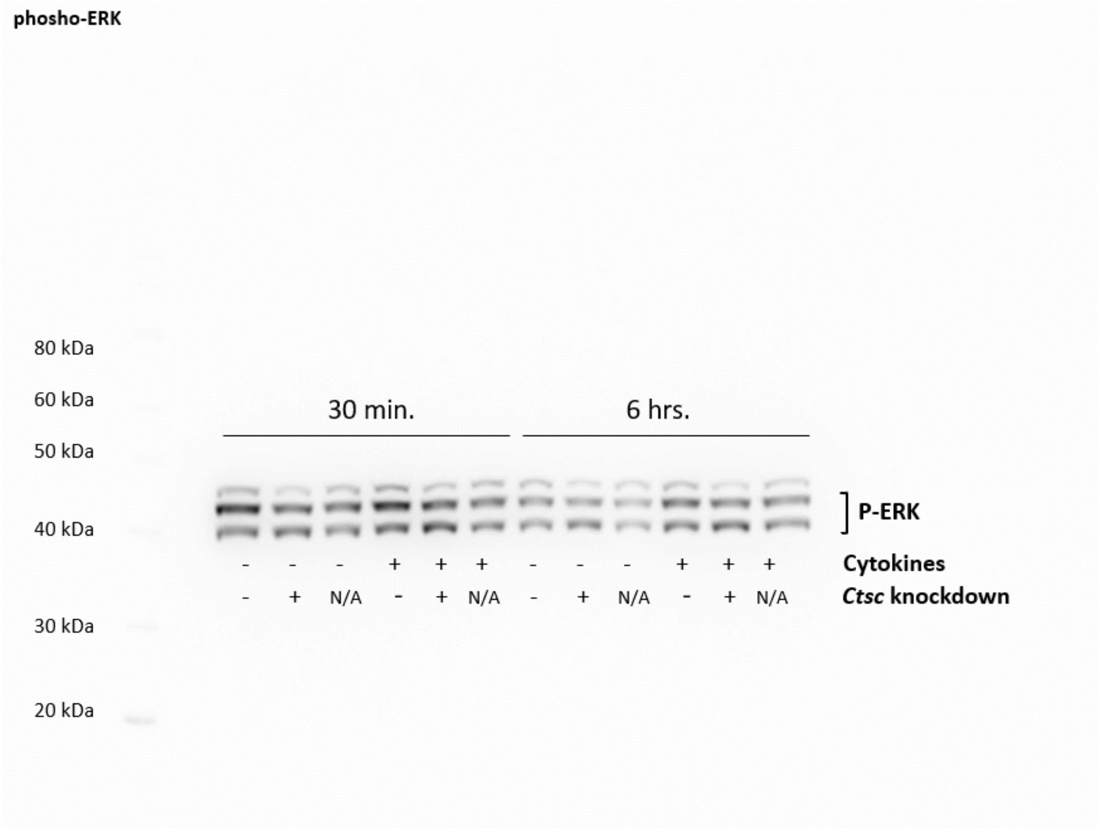
phosho-ERK
80 kDa
60 kDa
50 kDa
40 kDa
30 kDa
20 kDa
30 min.
6 hrs.
P-ERK
- - - + + + - - - + + + Cytokines
- + N/A - + N/A - + N/A - + N/A Ctsc knockdown

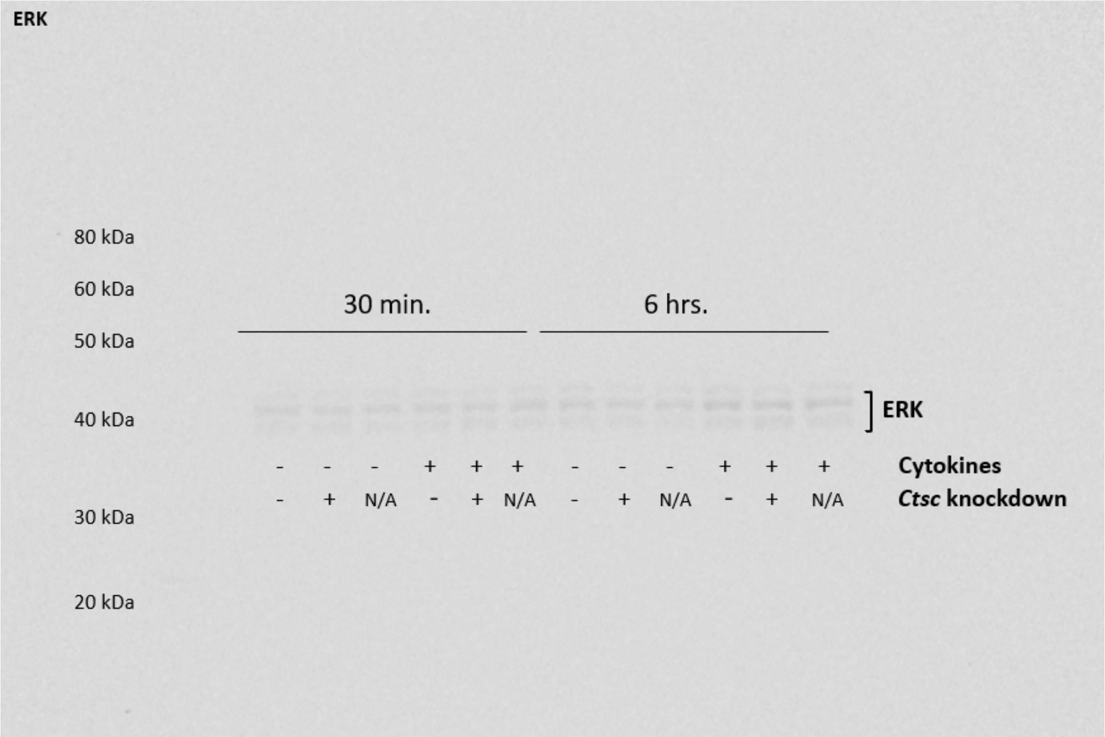
ERK
80 kDa
60 kDa
50 kDa
40 kDa
30 kDa
20 kDa
30 min.
6 hrs.
ERK
- - - + + + - - - + + + Cytokines
- + N/A - + N/A - + N/A - + N/A Ctsc knockdown

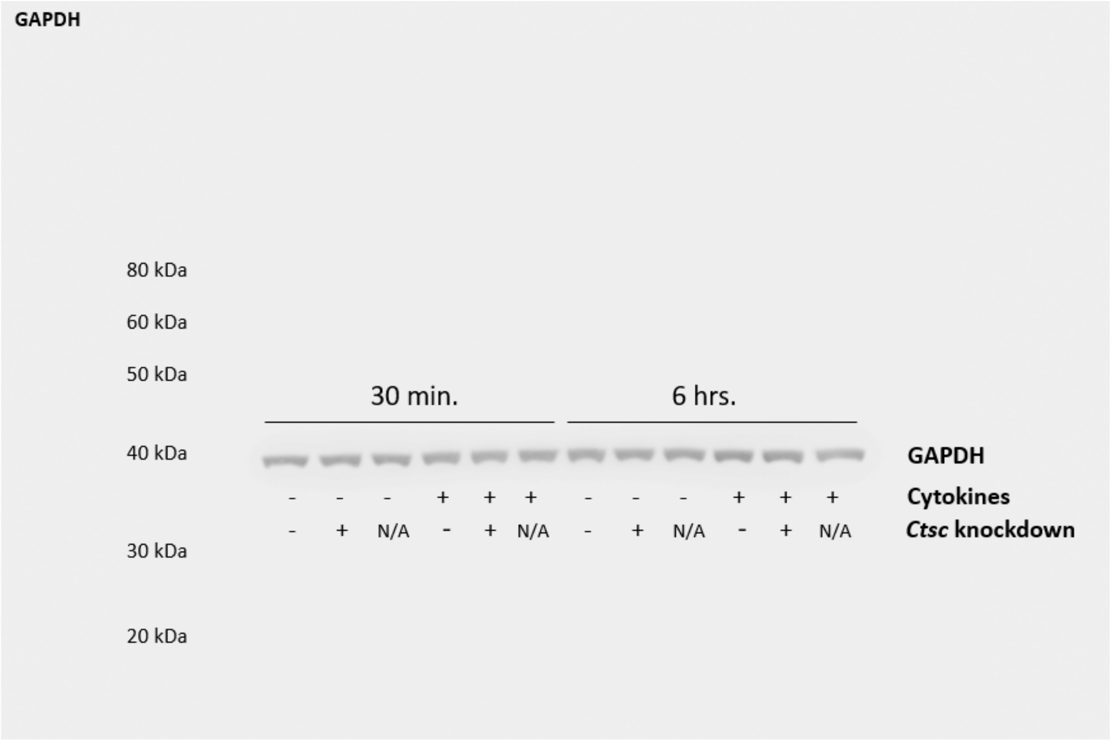
GAPDH
80 kDa
60 kDa
50 kDa
40 kDa
30 kDa
20 kDa
30 min.
6 hrs.
GAPDH
Cytokines
Ctsc knockdown
- - - + + + - - - + + +
- + N/A - + N/A - + N/A - + N/A

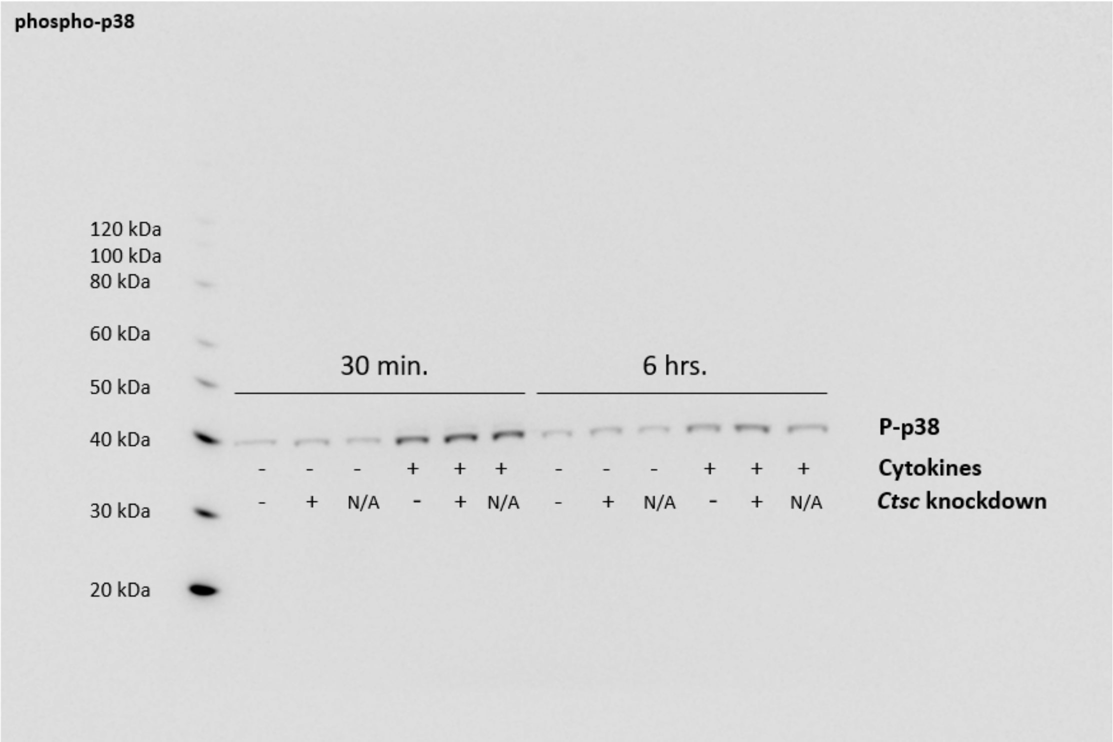
phospho-p38
120 kDa
100 kDa
80 kDa
60 kDa
50 kDa
40 kDa
30 kDa
20 kDa
30 min.
6 hrs.
P-p38
Cytokines
Ctsc knockdown
- - - + + + - - - + + +
- + N/A - + N/A - + N/A - + N/A

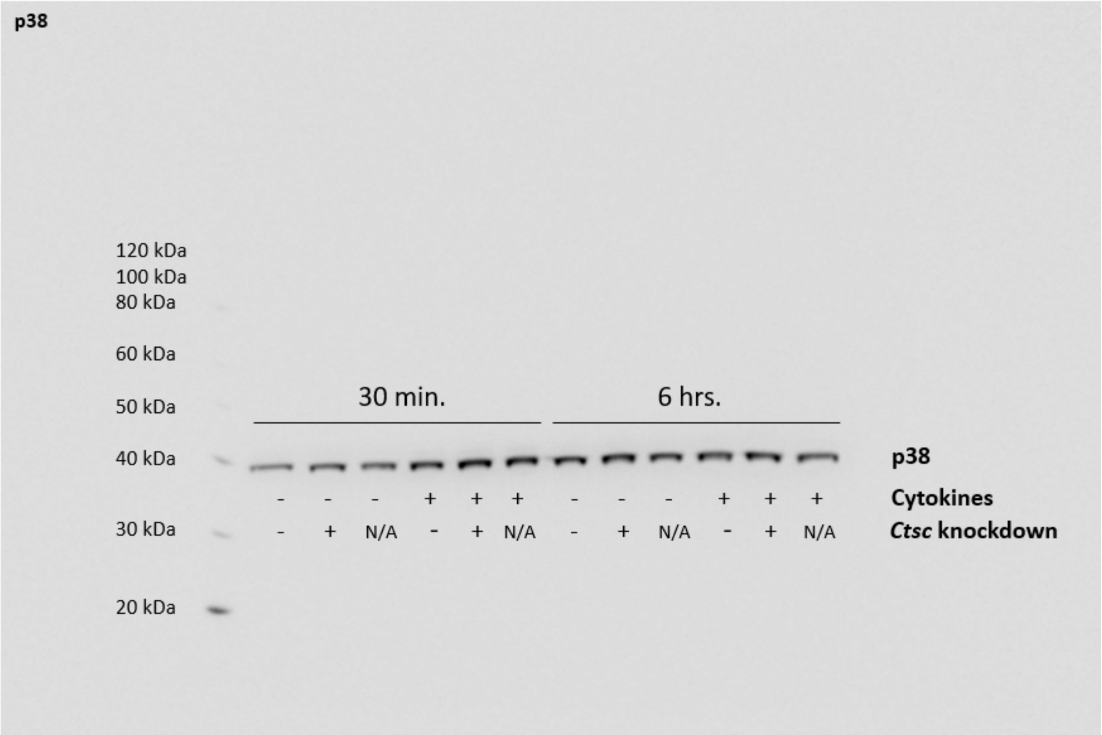
p38
120 kDa
100 kDa
80 kDa
60 kDa
50 kDa
40 kDa
30 kDa
20 kDa
30 min.
6 hrs.
p38
- - - + + + - - - + + +
Cytokines
- + N/A - + N/A - + N/A - + N/A
Ctsc knockdown

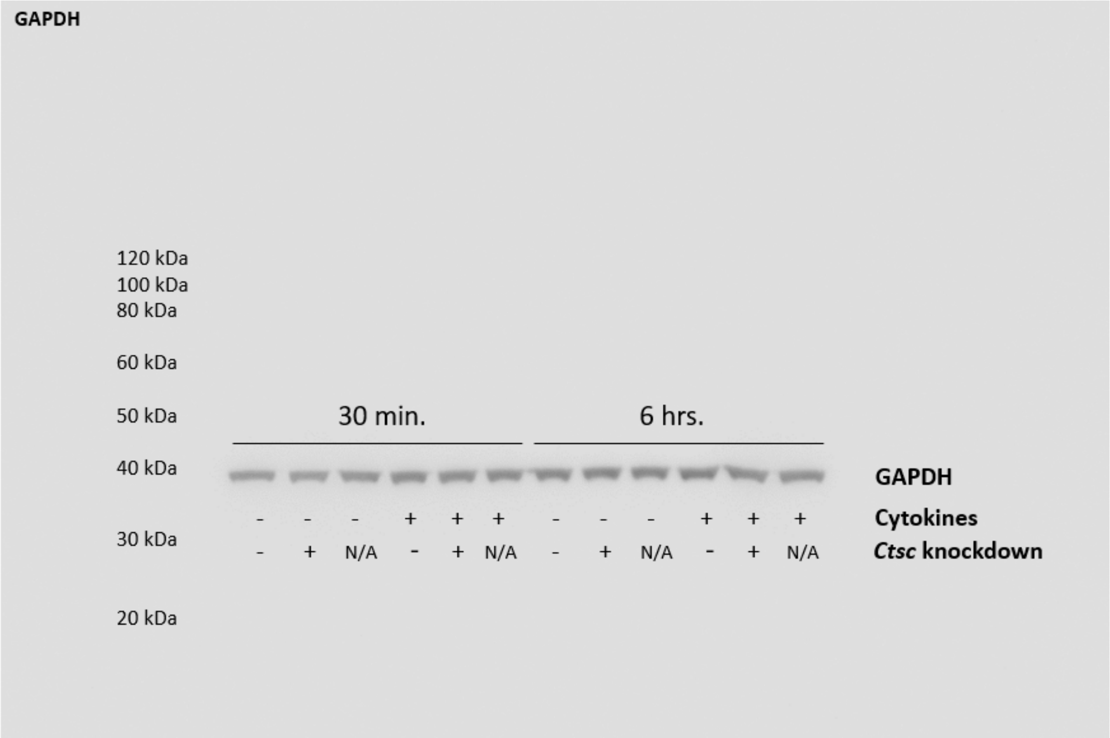
GAPDH
120 kDa
100 kDa
80 kDa
60 kDa
50 kDa
40 kDa
30 kDa
20 kDa
30 min.
6 hrs.
GAPDH
- - - + + + - - - + + +
Cytokines
- + N/A - + N/A - + N/A - + N/A
Ctsc knockdown

iNOS

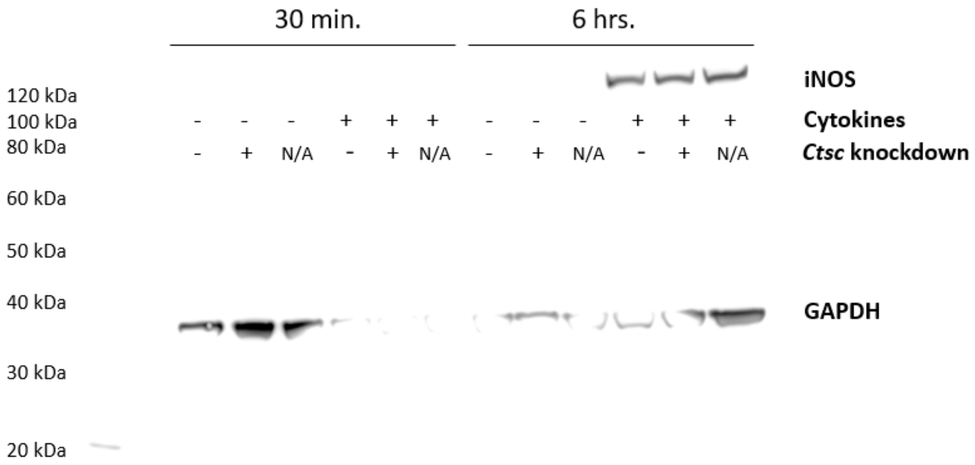

GAPDH

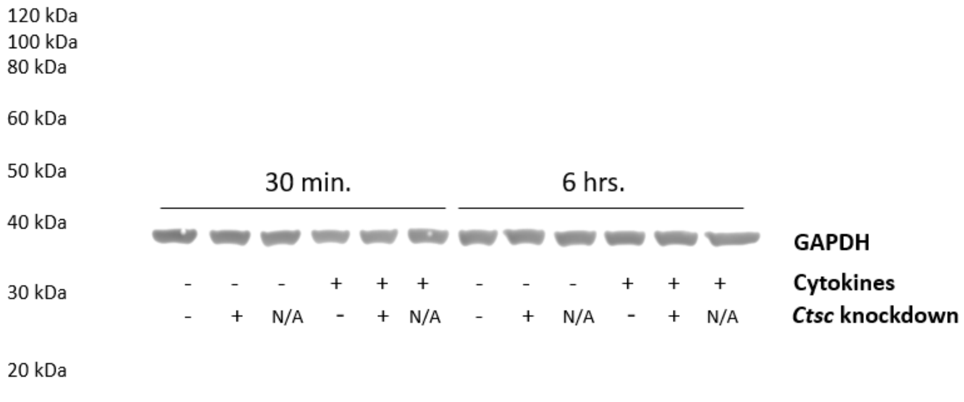

Supplementary Figure S5. Whole western blots of cleaved caspase-3, the MAPKs and iNOS upon *Ctsc* KD in INS-1E cells. Immunoblotting of cleaved caspase-3, phosphorylated and total JNK, ERK and p38 and iNOS in INS-1E cells transfected with siRNA against *Ctsc* or a non-targeting negative control siRNA, untreated or exposed to IL-1β and IFN-γ for 30 min and 6 h. Gapdh was used as loading control.
