# Supplementary material for: Cathepsin C Regulates Cytokine-Induced Apoptosis in β-Cell Model Systems
Source: Genes (Basel). 2021 Oct 25;12(11):1694. doi: 10.3390/genes12111694 (PMC8622156; doi:10.3390/genes12111694)
Supplement: Supplementary file 1 [file genes-12-01694-s001.zip › Supplementary Table S1.pdf]

Supplementary Table S1. Pancreatic islet donor information. Donor characteristics and human pancreatic islet specifications: unique identifier, gender: male (M)/ female (F), age (years), BMI (kg/m^2), HbA1c (%), origin/source, isolation centre, diabetes history, cause of death. islet viability (%) and purity (%).

**Islet preparation**	**1**	**2**	**3**	**4**	**5**
**Unique identifier**	HP-18144-01	HP-18151-01	HP-18270-01	HP-18304-01	HP-19256-01
**Gender (M/F)**	M	F	M	M	M
**Donor age (years)**	47	57	31	21	64
**BMI (kg/m^2)**	28.1	24.1	22.0	27.3	25.5
**HbA1c (%)**	5.8	5.8	5.5	5.4	5.4
**Origin/source**	UNOS	UNOS	UNOS	UNOS	UNOS
**Isolation centre**	Prodo Labs	Prodo Labs	Prodo Labs	Prodo Labs	Prodo Labs
**Diabetes history**	No	No	No	No	No
**Cause of death**	Head trauma	Stroke	Anoxic event	Head trauma	Stroke
**Islet viability (%)**	95	95	95	95	95
**Islet purity (%)**	85	90	90-95	85	90-95
